# IDH2 Deficiency Promotes Endothelial Senescence by Eliciting miR-34b/c-Mediated Suppression of Mitophagy and Increased ROS Production

**DOI:** 10.3390/antiox12030585

**Published:** 2023-02-27

**Authors:** Ikjun Lee, Shuyu Piao, Seonhee Kim, Harsha Nagar, Su-jeong Choi, Minsoo Kim, Giang-Huong Vu, Byeong-Hwa Jeon, Cuk-Seong Kim

**Affiliations:** Department of Physiology & Medical Science, School of Medicine, Chungnam National University, Daejeon 35015, Republic of Korea

**Keywords:** cellular senescence, endothelial cells, IDH2, mitophagy, miR-34b/c

## Abstract

Endothelial senescence impairs vascular function and thus is a primary event of age-related vasculature diseases. Isocitrate dehydrogenase 2 (IDH2) plays an important role in inducing alpha-ketoglutarate (α-KG) production and preserving mitochondrial function. However, the mechanism and regulation of IDH2 in endothelial senescence have not been elucidated. We demonstrated that downregulation of IDH2 induced accumulation of miR-34b/c, which impaired mitophagy and elevated mitochondrial reactive oxygen species (ROS) levels by inhibiting mitophagy-related markers (PTEN-induced putative kinase 1 (PINK1), Parkin, LC-II/LC3-I, and p62) and attenuating Sirtuin deacetylation 3 (Sirt3) expression. The mitochondrial dysfunction induced by IDH2 deficiency disrupted cell homeostasis and the cell cycle and led to endothelial senescence. However, miR-34b/c inhibition or α-KG supplementation restored Sirt3, PINK1, Parkin, LC-II/LC3-I, p62, and mitochondrial ROS levels, subsequently alleviating endothelial senescence. We showed that IDH2 played a crucial role in regulating endothelial senescence via induction of miR-34b/c in endothelial cells.

## 1. Introduction

The vascular endothelium in the inner layer of blood vessels is a crucial component that modulates vascular homeostasis and circulation and regulates vascular tone, blood fluidity, and inflammatory responses [1,2]. The senescence of vascular endothelial cells plays a vital role in the pathological changes in age-associated cardiovascular diseases [3]. Different stimuli, such as oxidative stress, inflammation, and genomic stress, alter the morphology and permeability of the endothelium and contribute to the development and progression of endothelial senescence [4]; however, the mechanisms underlying endothelial senescence have not been fully elucidated.

Endothelial senescence is associated with the accumulation of dysfunctional mitochondria, including altered mitochondrial dynamics, decreased mitochondrial mass, and impaired mitochondrial biogenesis [5,6]. Moreover, damaged mitochondria generate excessive reactive oxidative species (ROS) and elicit a proinflammatory phenotype, subsequently contributing to endothelial senescence. In the mitochondria, α-ketoglutarate (α-KG), converted from isocitrate by isocitrate dehydrogenase 2 (IDH2), is a key endogenous metabolite in the tricarboxylic acid (TCA) cycle. α-KG plays an important role in regulating ATP production and ROS modulation [7]. Shahmirzadi and colleagues revealed that the level of α-KG declined with age, and α-KG supplementation extended the lifespan and reduced morbidity [8]. In addition, mitochondrial function is gradually impaired during aging, contributing to a reduction in mitochondrial metabolic flux and decreased α-KG levels. IDH2 catalyzes the production of α-KG, and our previous study demonstrated that IDH2 downregulation led to mitochondrial dysfunction accompanied by excessive ROS, elevated inflammation, and disrupted mitochondrial dynamics in endothelial cells [9,10]. We aimed to establish whether IDH2 plays a role in the decreased α-KG level in aging and explored the internal mechanism of IDH2 and endothelial senescence.

## 2. Materials and Methods

### 2.1. Reagents

Human umbilical vein endothelial cells (HUVECs) at passages 2–9 were purchased from Clonetics (San Diego, CA, USA) and cultured in endothelial growth medium 2 at 37 °C in a 5% CO_2_ incubator according to the manufacturer’s instructions. Small interfering RNA (siRNA) was used for gene knockout. Either negative control or IDH2-specific siRNA (Bioneer, Daejeon, Republic of Korea) or a miR-34b/c mimic (cosmoGENETECH, Seoul, Republic of Korea) was transfected into HUVECs using Lipofectamine 2000 (Invitrogen, Carlsbad, CA, USA) for 48 h according to the manufacturer’s recommendations. After 24 h of transfection, the cells were treated with the miR-34b/c inhibitor (cosmoGENETECH) or α-KG (Sigma-Aldrich, St. Louis, MO, USA) for 24 h.

### 2.2. Mouse Study

All animal experiments were performed following the guidelines of the Institutional Animal Care and Use Committee of Chungnam National University (CNUH-015-A0015-2). IDH2-knockout (IDH^−/−^ germ-line knockout) mice were a generous donation from Kyungpook National University (School of Life Sciences, College of Natural Science, Daegu, Republic of Korea). ALZET^®^ osmotic pumps (DURECT, Cupertino, CA, USA) containing scrambled miRNA or the miR-34b/c inhibitor were implanted into wild-type (WT) or IDH2-knockout mice for 2 weeks for in vivo infusion of the miRNA inhibitor.

### 2.3. Endothelial Cell Isolation

Lungs from young (1~2 months old) and old (>18 months old) WT and IDH2-knockout (8~10 weeks old) mice were used to isolate endothelial cells. Each mouse was anesthetized by urethane (1.3 g/kg, intraperitoneal injection), and the lungs were removed. The lungs were incubated with Dulbecco’s Modified Eagle’s Medium (Gibco, Grand Island, NY, USA) with 1 mg/mL collagenase type I (Worthington, Lakewood, NJ, USA) for 40 min at 37 °C with gentle shaking. The digested lungs were filtered through a 100 μm filter and briefly centrifuged for 5 min at 700× *g*. After removing red blood cells using red blood cell lysis buffer (Thermo-Fisher Scientific, Waltham, MA, USA), the cells were washed with phosphate-buffered saline (PBS). Endothelial cells were isolated from the single-cell suspensions using Magnetic-Activated Cell Sorting (Miltenyi Biotec Inc., Bergisch Gladbach, Germany) performed with anti-CD45 and anti-CD31 antibody-conjugated magnetic beads according to the manufacturer’s instructions (CD45 bead, cat no. 130-052-301; CD31 bead, cat no. 130-097-418. Miltenyi Biotec Inc., Bergisch Gladbach, Germany). CD45-negative and CD31-positive cells were isolated from the cell population.

### 2.4. Immunoblotting

HUVECs and lung endothelial cells were lysed with radio-immunoprecipitation assay (RIPA) buffer (Cell Signaling Technology, Danvers, MA, USA), sonicated, and centrifuged. The supernatants were harvested, and the protein concentration was measured using a bicinchoninic acid (BCA) kit (Thermo-Fisher Scientific); 20 μg of the lysate was analyzed as described previously [8]. The following primary antibodies were purchased: p16, p21, p62, and Sirt3 antibodies from Santa Cruz Biotechnology (Santa Cruz, CA, USA); p53, p-RB, t-RB, PINK1, Parkin, Myc, and IDH2 antibodies from Abcam (Cambridge, UK); LC3 antibody from NOVUS Biologicals (Centennial, CO, USA); COX IV antibody from cell signaling (4844s); and p62 and β-actin antibodies from Sigma-Aldrich. Mouse and rabbit secondary antibodies were obtained from Thermo-Fisher Scientific (Waltham, MA, USA).

### 2.5. Real-Time Quantitative Polymerase Chain Reaction

Total mRNA was obtained from HUVECs or lung endothelial cells using TRIzol reagent (Thermo-Fisher Scientific, Waltham, MA, USA) following the manufacturer’s protocols. After measuring the mRNA concentration, cDNA or miRNA was generated using cDNA synthesis master mix (CellSafe, Yongin, Republic of Korea) or the miScript kit (Qiagen, Hilden, Germany), respectively, according to the manufacturer’s instructions. The StepOnePlus Real-Time PCR system (Applied Biosystems, Foster City, CA, USA) with SYBR green qPCR premix (Enzynomics, Daejeon, Republic of Korea) was used for the analysis. See Supplementary Table S1 for the primer sequences used for quantification. IDH2 and β-actin primers were purchased from Bioneer. β-Actin or RNU6 was used as an internal control, and relative expression was calculated using the 2^−Δ/ΔCt^ method.

### 2.6. Plasmid Transfection

Plasmids containing the full-length WT or mutated IDH2 sequence were used to evaluate the effects of IDH2 expression on Myc-tagged Sirt3, PINK1, and Parkin levels. Sirt3, PINK1, and Parkin plasmid were purchased from Sino Biological (Beijing, China), and the sequence-mutated plasmid, which mismatched with the miR-34b/c seed sequence, was produced by cosmoGENETECH (Supplementary Figure S4). The negative control or miR-34b/c mimic was first transfected into HEK293T cells using Lipofectamine 2000 for 24 h, and then the WT or mutated IDH2 plasmid was transfected into the cells using Effectene transfection reagent (Qiagen) for another 24 h. Myc expression was used as a tag to assess the binding affinity of miR-34b/c to Sirt3, PINK1, and Parkin.

### 2.7. Flow Cytometry

The cell cycle of HUVECs was analyzed using the NovoCyte flow cytometer (ACEA Biosciences, San Diego, CA, USA). After transfection, cells were washed with PBS and fixed with ice-cold 70% ethanol overnight at 4 °C. The cells were washed twice with PBS to remove the ethanol before staining with PI (5 μg/mL) with RNase A (1 mg/mL) for 30 min at 37 °C. NovoExpress software (ACEA Biosciences, San Diego, CA, USA) was used to analyze the flow cytometry data.

### 2.8. Senescence-Associated Beta-Galactosidase (SA-β-gal) Staining

HUVECs or aortas from WT and IDH2 mice were washed with PBS and fixed with 4% formaldehyde overnight at 4 °C. After removing the formaldehyde, the cells or aortas were rewashed and fixed using the SA-β-galactosidase staining kit (Sigma-Aldrich) according to the manufacturer’s protocol.

### 2.9. Measurement of Mitochondrial ROS

Mitochondrial ROS levels in HUVECs or endothelial cells from the mice were measured using MitoSOX red fluorescent dyes. HUVECs (1 × 104/well) were seeded on 12 well glass bottom plates and incubated overnight. After transfection with NC or IDH2 siRNA for 48 h, HUVECs were incubated in 0.1 μM MitoTracker Green solution (MT-10, Dojindo, Kumamoto, Japan) in EGM2 at 37 °C for 30 min in the dark. After removing the supernatant, cells were washed twice with Hank’s Balanced Salt Solution (HBSS) and then incubated in 10 μM mitoSOX solution (MT-14, Dojindo, Kumamoto, Japan) with Antimycin A (5 μM) or carbonyl cyanide m-chlorophenyl hydrazone (CCCP) (20 μM), or only mitoSOX in EGM2 at 37 °C for 30 min in the dark. After washing once with HBSS, cells were mounted using Vectashield Antifade Mounting Medium with 4,6-diamidino-2phenylindole (DAPI). The stained cells were imaged using a fluorescence microscope (Axio Vert.A1; Zeiss, Heidelberg, Germany). MitoTracker Green image was observed in the green channel (Ex: 488 nm, Em: 500~550 nm), MitoSOX Red image was observed in the red channel (Ex: 633 nm, Em: 640~700 nm). In addition, isolated HUVECs or lung endothelial cells were also incubated with 5 μM MitoSOX at 37 °C for detection of fluorescence intensity. Fluorescent signals were also measured using the Fluoroskan Ascent fluorescence reader (Thermo-Fisher Scientific).

### 2.10. Measurement of α-KG, Citrate, and Glutamine Content

α-Ketoglutarate assay kit (Cat: K677-100, Biovision, Milpitas, CA, USA), citrate assay kit (Cat: K655-100, Biovision, Milpitas, CA, USA), and glutamine assay kit (Cat: K556-100, Biovision, Milpitas, CA, USA) were used to measure the intracellular α-KG, citrate, and glutamine level, respectively. Isolated HUVECs or endothelial cells were resuspended in an assay buffer. Following sonication and centrifugation, the supernatants were used to analyze the α-KG, citrate, and glutamine level according to the manufacturer’s instructions.

### 2.11. Measurement of Isocitrate Dehydrogenase (IDH) Activity

IDH activity was measured using a commercial IDH colorimetric assay kit (Cat: ab102528, Abcam, Cambridge, MA, USA). Briefly, isolated endothelial cells were resuspended in 200 assay ul ice-cold buffer. Following sonication and centrifugation (13,000× *g*, 10 min), the supernatants were used to analyze the IDH activity according to the manufacturer’s instructions. NADP^+^ was added to the isocitrate substrate for the determination of NADP^+^-dependent IDH isoforms.

### 2.12. Statistical Analysis

Statistical analyses were performed using GraphPad Prism 8 software (GraphPad Software, La Jolla, CA, USA). Student’s *t*-test was used to evaluate the differences between the two groups. For multiple comparisons, one-way analysis of variance (ANOVA) was performed following Tukey’s post hoc test, and two-way ANOVA was used following Sidak’s post hoc testing. Data are presented as means ± SEM. A value of *p* ≤ 0.05 indicated statistical significance. All data are representative of at least three independent experiments.

## 3. Results

### 3.1. Endothelial IDH2 Expression Decreased during Aging and Was Related to Senescence Markers

**Figure 1:**
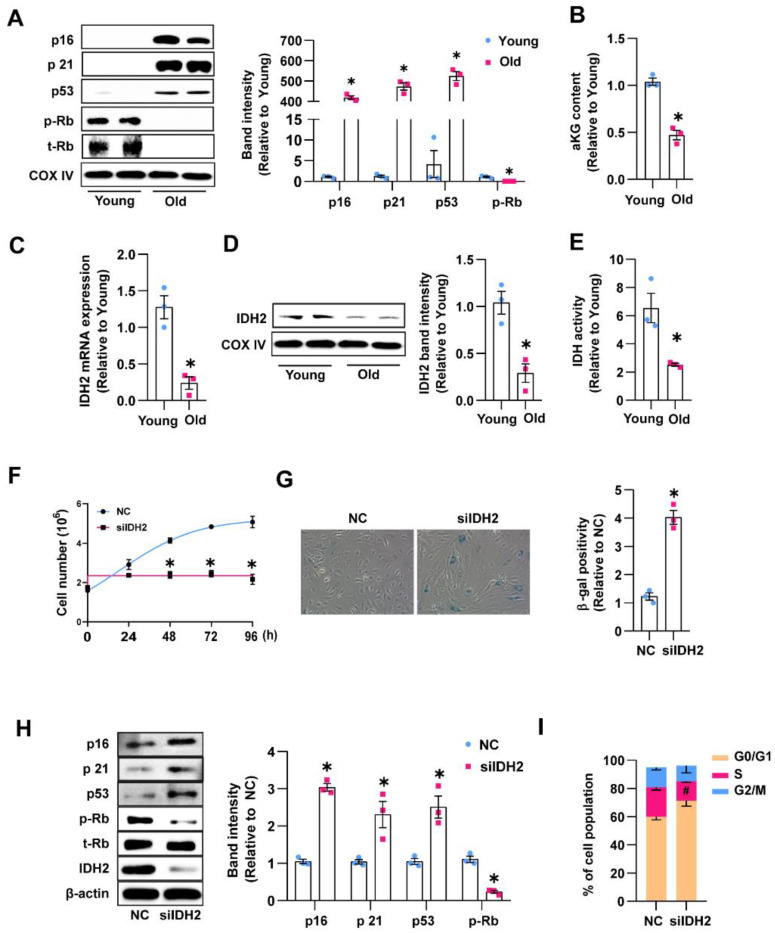
IDH2 downregulation contributes to senescence of endothelial cell. (**A**) Expression of cell cycle regulators in lung endothelial cells from young and old mice. (**B**) Intracellular α-KG levels of young and old mice group. (**C**,**D**) IDH2 expression in young and old mice analyzed by qPCR and Western blot. (**E**) IDH activity in lung endothelial cells from young and old mice. (**F**) Counting of cell growth for 24–96 h after NC (negative control) or siIDH2 (siRNA against IDH2) transfection. Logistic growth curve was drawn, and statistical analysis was used by two-way ANOVA. (**G**) SA-β-gal staining in cell with NC or siIDH2 for 48 h (20× magnification). (**H**) Western blot analysis of cell cycle regulators. (**I**) Analysis of cell cycle by flow cytometry. *n* = 3 per group, mean ± SEM, * *p* < 0.05 compared with young or NC-treated cells. # *p* < 0.05 compared with NC-treated cells.

As a critical metabolite of the TCA cycle, α-KG affects crucial elements of aging and enhances longevity [8]. We investigated the α-KG level in the endothelial cells of aging mice. First, we evaluated the levels of cell cycle regulatory proteins to confirm that the characteristics of senescent endothelial cells were already developed in aging mice (18 months old). Lung endothelial cells from aged mice showed markedly higher levels of cell cycle regulatory proteins (p16, p21, and p53) and a lower level of phosphorylated retinoblastoma (RB) (Figure 1A). Subsequently, we measured the α-KG level in endothelial cells from young (2 months old) and aged mice (18 months old), and as expected, the cellular α-KG level was significantly lower in aged mice than young mice (Figure 1B). As IDH2 is a key metabolic enzyme that converts isocitrate to α-KG via the TCA cycle, we evaluated IDH2 expression in lung endothelial cells. The mRNA and protein levels of IDH2 were markedly lower in aged mice than in young mice (Figure 1C,D). Moreover, the IDH activity in aged mice is also evidently decreased compared to young mice (Figure 1E). To further investigate the role of IDH2 in endothelial senescence in vitro, we knocked out IDH2 by siRNA transfection in HUVECs and then monitored their proliferation and levels of other cellular senescence markers. From 48 h after transfection, IDH2-knockout cells significantly decreased in cell proliferation compared with the control siRNA-transfected cells (Figure 1F). IDH2-knockout cells exhibited an enlarged and flattened cell morphology and increased SA-β-gal expression compared with the control cells (Figure 1G). The expression of cell cycle regulatory proteins was evaluated to clarify the underlying cell cycle mechanisms. IDH2 knockout elevated the protein levels of p16, p21, and p53 and inhibited RB phosphorylation, indicating cell cycle arrest (Figure 1H). Fluorescence-activated cell sorting analysis showed that IDH2 knockout resulted in significant cell growth arrest by increasing the cell population in the G0/G1 phase and decreasing that in the S phase (Figure 1I). These results suggested that endothelial IDH2 expression is decreased in aging mice and that IDH2 deficiency accelerates endothelial senescence in HUVECs.

### 3.2. miR-34b/c Regulated the Expression of sirt3, PINK1, and Parkin in IDH2-Knockout Cells

**Figure 2:**
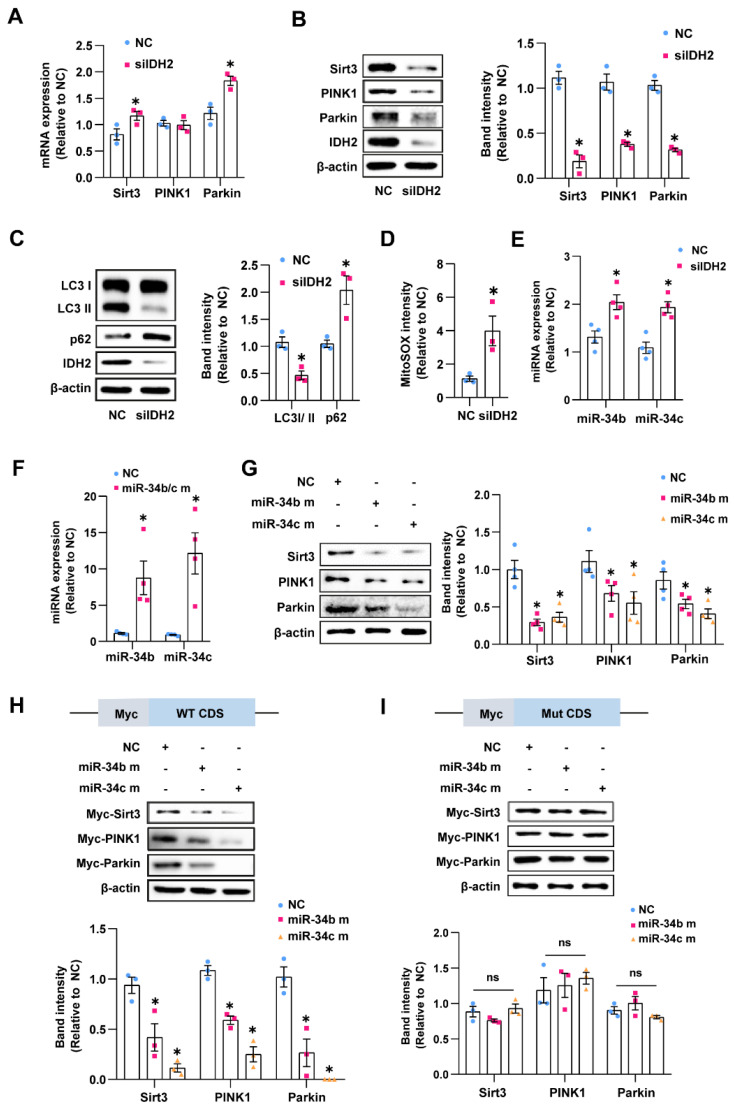
miR-34b/c regulated the expression of Sirt3, PINK1, and Parkin in IDH2-knockout cells: (**A**,**B**) Sirt3, PINK1, and Parkin expression in NC or siIDH2 group analyzed by qPCR and Western blot. (**C**) Western blot analysis of LC3 and p62 in HUVECs with NC or siIDH2. (**D**) Mitochondrial ROS analysis by Mito-SOX fluorescence intensity in NC or siIDH2-transfected cells. (**E**) miR-34b/c mRNA levels in cells by qPCR. (**F**) Induction of miR-34b/c through mimic transfection. (**G**) Induction of miR-34b/c suppressed Sirt3, PINK1, and Parkin quantified by Western blot. (**H**,**I**) Analysis of affinity between miR-34b/c and Sirt3-PINK1-Parkin using WT and mutated plasmid by Western blot-mediated Myc quantification in HEK293T. (WT CDS: wild type coding sequence; Mut CDS: mutation coding sequence) *n* = 3 per group, mean ± SEM, ns: no statistical significance * *p* < 0.05 compared with NC-treated cells.

Previous studies showed that Sirt3 is associated with aging-related diseases and is crucial in regulating mitochondrial metabolism and homeostasis [11,12,13]. We previously showed that IDH2 downregulation induced mitochondrial dysfunction by disrupting mitochondrial dynamics and increasing mitochondrial oxidative stress [9]. PINK1/Parkin-regulated mitophagy preserves mitochondrial function and concomitantly prevents endothelial senescence [14,15]. Therefore, we investigated the mRNA and protein levels of Sirt3, PINK1, and Parkin in IDH2-knockout HUVECs. Sirt3 and Parkin mRNA levels were increased in IDH2- knockout cells (Figure 2A). However, the knockout of IDH2 resulted in decreased Sirt3, PINK1, and Parkin protein levels, which was inconsistent with the mRNA levels (Figure 2B). The reduced PINK1 and Parkin protein levels were accompanied by decreased mitophagy flux (reduced LC-II/LC3-I ratio and accumulated p62 expression) (Figure 2C). Either Sirt3 suppression or inhibition of mitophagy can trigger mitochondrial ROS production via reduced deacetylation of manganese superoxide dismutase and accumulation of damaged mitochondria. IDH2 deficiency significantly elevated mitochondrial ROS levels (Figure 2D and Figure S3). Previous studies showed that miRNAs suppress protein expression by inhibiting translation and promoting mRNA degradation [16]. The miR-34 family can also alter autophagic flux and suppress cell proliferation [17,18,19]. Therefore, we investigated whether miR-34b/c plays a role in the discrepancy between the mRNA and protein levels of Sirt3, PINK1, and Parkin. IDH2 knockout markedly increased miR-34b/c expression compared with the control cells (Figure 2E). We overexpressed miR-34b/c via transfection of a miR-34b/c mimic to explore the role of miR-34b/c in regulating the protein expression of Sirt3, PINK1, and Parkin. There was a robust increase in miR-34b/c expression after transfection of the mimic.

Figure 2G shows a marked decrease in the protein levels of Sirt3, PINK1, and Parkin compared with the control group (Figure 2G). Next, we performed a TargetScan v.7.1 analysis of miR-34b/c-binding sites within Sirt3, PINK1, and Parkin mRNA. We predicted that miR-34b/c targets the 3′ untranslated region of Sirt3 and the protein-coding regions of PINK1 and Parkin (Supplementary Figure S4B). To validate whether these regions of Sirt3, PINK1, and Parkin are genuine targets of miR-34b/c, we expressed Myc-tagged WT Sirt3, PINK1, and Perkin, or their mutant versions in HEK293T cells. The mutant version had a mutation in their sequence that matched with the miR-34b/c seed sequence (Supplementary Figure S4C–E). Co-transfection of the miR-34b/c mimic with the WT c-Myc plasmid significantly suppressed Sirt3, PINK1, and Parkin expression. However, co-transfection of the miR34b/c mimic with the mutant c-Myc plasmid did not inhibit Sirt3, PINK1, or Parkin expression (Figure 2H,I), suggesting that miR34b/c binds directly to the predicted regions of Sirt3, PINK1, and Parkin to prevent their protein expression. These findings confirmed that miR-34b/c inhibited the expression of Sirt3, PINK1, and Parkin in HUVECs in a site-specific manner.

### 3.3. miR-34b/c Mediated the Endothelial Senescence Induced by IDH2 Knockout by Inhibiting Mitophagy and Elevating Mitochondrial ROS

**Figure 3:**
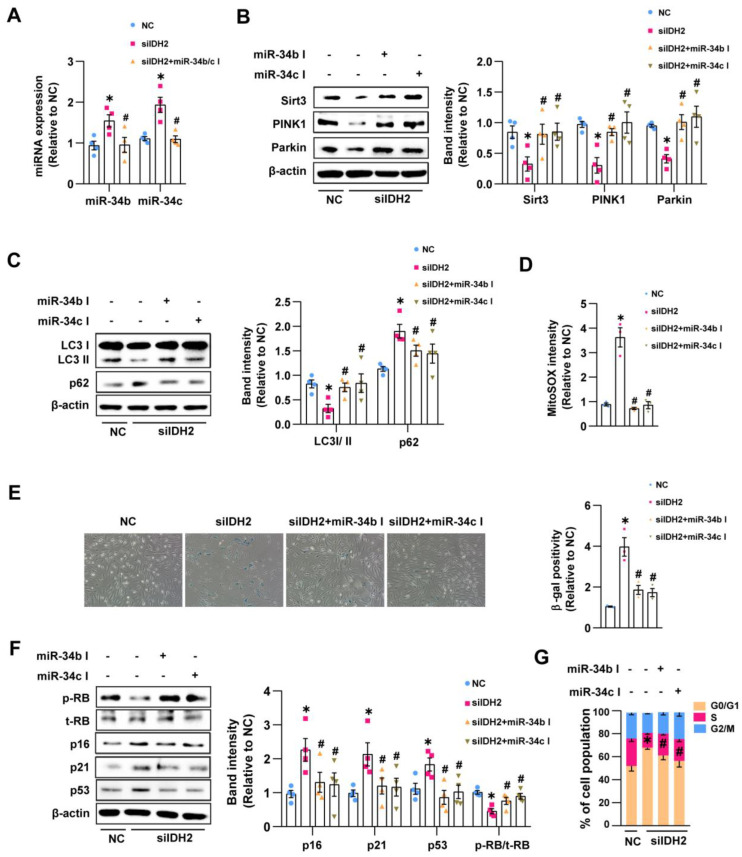
miR-34b/c mediated the endothelial senescence induced by IDH2 knockout by inhibiting mitophagy and elevating mitochondrial ROS: (**A**) Alteration of miR-34b/c contents by miR-34b/c specific inhibitor by qPCR. (**B**) Western blot analysis of Sirt3, PINK1, and Parkin expression after siIDH2 and miR-34b/c inhibitor transfection. (**C**) Quantification of LC3 II/I ratio and p62 using Western blot (**D**) Analysis of mtROS by measuring MitoSOX intensity. (**E**) Representative images and quantification of the SA-β-gal positive cells (20×). (**F**) Densitometrical measurement of cell cycle regulators by Western blot. (**G**) Analysis of cell portion of each cell cycle phases using flow cytometry. *n* ≥ 3 per group, mean ± SEM, * *p* < 0.05 compared with NC-treated cells, # < 0.05 compared with siIDH2-treated cells.

We demonstrated that miR-34b/c activation decreased the expression of Sirt3, PINK1, and Parkin. Thus, we evaluated whether miR-34b/c activation contributes to the decreased Sirt3, PINK1, and Parkin expression in IDH2-knockout cells. First, we confirmed that the miR-34b/c inhibitor efficiently reduced IDH2-knockout-induced miR-34b and miR-34c levels (Figure 3A). We then evaluated Sirt3 and mitophagy-related protein expression. The miR-34b/c inhibitor prevented the decreases in the Sirt3, PINK1, Parkin, and LC3 II/I protein levels and an increase in the p62 level in IDH2-knockout cells (Figure 3B,C). The miR-34b/c inhibitor also reduced mitochondrial ROS levels in IDH2-knockout cells compared with untreated IDH2-knockout cells (Figure 3D). Therefore, IDH2 deficiency inhibited mitophagy and elevated mitochondrial ROS levels via miR-34b/c-suppressed Sirt3, PINK1, and Parkin expression. Subsequently, we evaluated whether the recovery of Sirt3, PINK1, and Parkin expression alleviates IDH2-knockout-induced endothelial senescence in HUVECs. We showed that inhibition of miR-34b/c attenuated SA-β-gal expression (Figure 3E), reduced cell cycle regulator (p16, p21, and p53) expression, and enhanced RB phosphorylation compared with the untreated IDH2-knockout cells (Figure 3F). Additionally, the miR-34b/c inhibitor decreased the population of cells in the G0/G1 phase. These results suggest that miR-34b/c plays a crucial role in IDH2-knockout-dependent endothelial senescence by preventing mitophagy and stimulating mitochondrial ROS production.

### 3.4. IDH2 Deficiency Activated miR-34b/c by Reducing the α-KG Level in HUVECs

**Figure 4:**
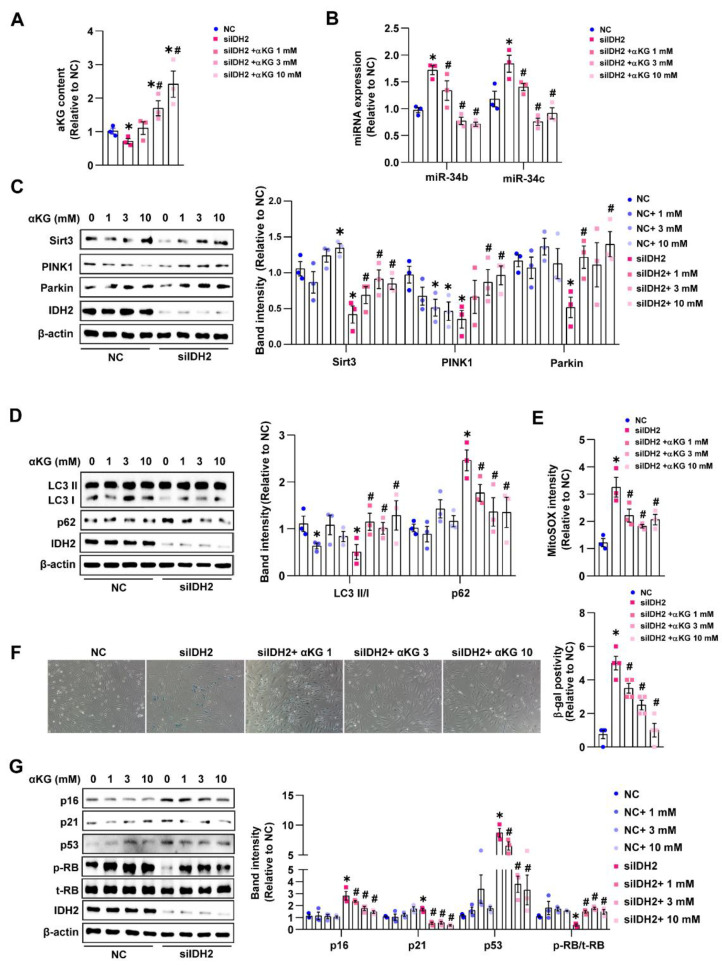
IDH2 deficiency activated miR-34b/c by reducing the α-KG level in HUVECs: (**A**) Intracellular α-KG levels measurement with concentration-dependent manner of α-KG in siIDH2 group. (**B**) Alteration of miR-34b or miR-34c levels by qPCR. (**C**) Quantification of Sirt3, PINK1, and Parkin expression in NC or siIDH2-treated cells with different amounts of α-KG by Western blot. (**D**) Western blot analysis of LC3 II/I ratio and p62 in NC or siIDH2 group with various concentrations of α-KG (**E**) mtROS measurement in siIDH2 and α-KG -treated cells. (**F**) SA-β- gal staining of HUVECs with NC or siIDH2 or siIDH2 plus various α-KG concentration. (**G**) Analysis of the expression of cell cycle regulators in NC or siIDH2-treated HUVECs with concentration-dependent manner of α-KG. *n* = 3 per group, mean ± SEM, * *p* < 0.05 compared with NC-treated cells with 0 mM α-KG, # < 0.05 compared with siIDH2 with 0 mM α-KG.

IDH2 reversibly catalyzes the conversion of isocitrate to α-KG. Considering the low levels of IDH2 and α-KG in endothelial cells from aging mice, we evaluated the expression of α-KG in vitro and its ability to regulate miR-34b/c activation. The cellular α-KG level was consistent with the in vivo results and was lower in IDH2-knockout HUVECs, and α-KG treatment in IDH2-knockout cells significantly increased its level (Figure 4A). Next, we pretreated IDH2-knockout cells with various concentrations of α-KG. The elevated miR-34b/c expression in the IDH2-knockout cells was significantly inhibited by α-KG treatment, indicating that the reduced α-KG level triggered by IDH2 knockout contributed to miR-34b/c activation (Figure 4B). Moreover, α-KG treatment rescued the expression of Sirt3 and the mitophagy-related proteins PINK1, Parkin, LC3 II/1, and p62 (Figure 4C,D), which in turn reduced mitochondrial ROS levels and cellular SA-β-gal activity and reduced the levels of senescence markers (including p16, p21, p53, and p-RB) in IDH2-knockout cells (Figure 4E–G). From these results, we concluded that a decrease in the α-KG level is crucial for promoting IDH2 knockout-induced mitophagy and endothelial senescence in vitro.

### 3.5. IDH2 Knockout Promoted Endothelial Senescence and Impaired Mitophagy Associated with miR-34b/c Activation In Vivo

**Figure 5:**
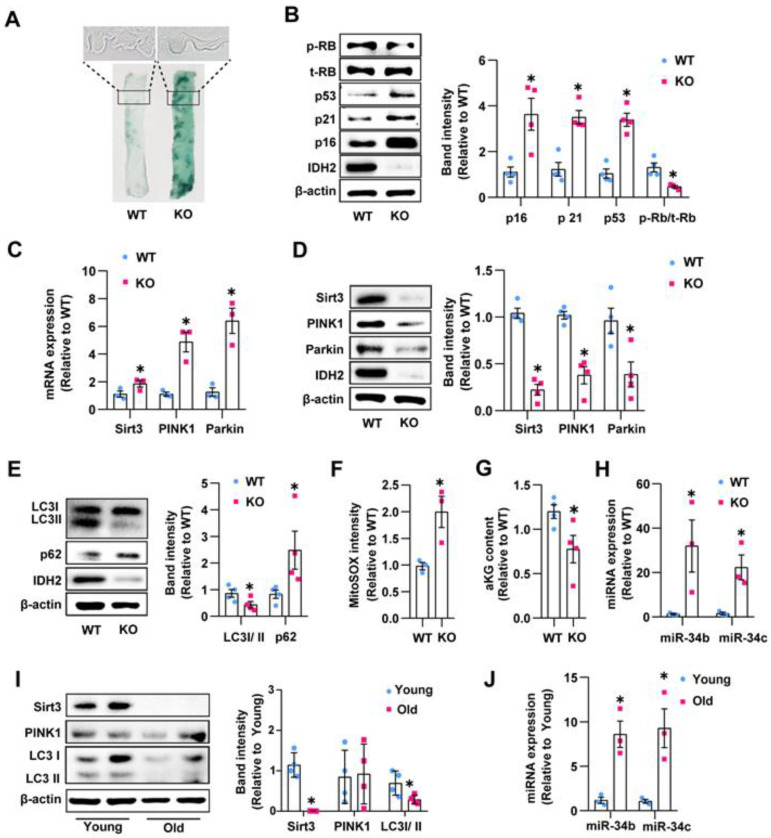
IDH2 knockout promoted endothelial senescence and impaired mitophagy associated with miR-34b/c activation in vivo: (**A**) SA-β-gal staining on endothelium surface from aorta of WT or IDH2 KO mouse (20X). (**B**) Western blot analysis of cell cycle regulars in endothelial cells from WT or IDH2 KO mice. (**C**,**D**) Quantification of Sirt3, PINK1, Parkin expression by qPCR and Western blot. (**E**) Analysis of LC3 II/I ratio and p62 by Western blot. (**F**) mtROS measurement using MitoSOX fluorescence intensity in endothelial cells. (**G**) Intracellular α-KG contents of WT and IDH2 KO mice. (**H**) Abundance of miR-34b/c in endothelial cells. (**I**) Western blot analysis of LC3 II/I ratio, PINK1, and Sirt3 in endothelial cells from young or old mice. (**J**) Abundance of miR-34b/c in endothelial cells from young or old mice. *n* ≥ 3 per group, mean ± SEM, * *p* < 0.05 compared with WT.

We performed SA-β-gal staining in aortic endothelial surface tissues from WT and IDH2-knockout mice to examine the effect of IDH2 deficiency on endothelial senescence in vivo. Aortas from IDH2-knockout mice showed enhanced SA-β-gal staining intensity compared with WT mice (Figure 5A). Additionally, we isolated endothelial cells from lung tissue and assessed the protein expression of p16, p21, and p53; cell cycle regulators; and RB phosphorylation. IDH2-knockout mice showed increased expression of p16, p21, and p53 and decreased phosphorylation of RB (Figure 5B). Sirt3, PINK1, and Parkin showed a corresponding decrease in mRNA expression and an increase in protein expression, consistent with the in vitro results (Figure 5C,D). IDH2 knockout also altered LC3 II/I and p62 levels and increased mitochondrial ROS levels in endothelial cells (Figure 5E,F). IDH2-knockout endothelial cells showed a decreased α-KG level and increased miR-34b/c level compared with WT mice (Figure 5G,H). Interestingly, endothelial cells in aged mice also showed reduced Sirt3 and LCII/I levels and an increased miR-34b/c level (Figure 5I,J); however, the expression of PINK1 did not differ between WT and IDH2-knockout aged mice (Figure 5I). Our results suggested that IDH2 knockout induced endothelial senescence and impaired mitophagy associated with elevated miR-34b/c and mitochondrial ROS levels in vivo.

### 3.6. Systemic Inhibition of miR-34b/c Ameliorated Endothelial Senescence

**Figure 6:**
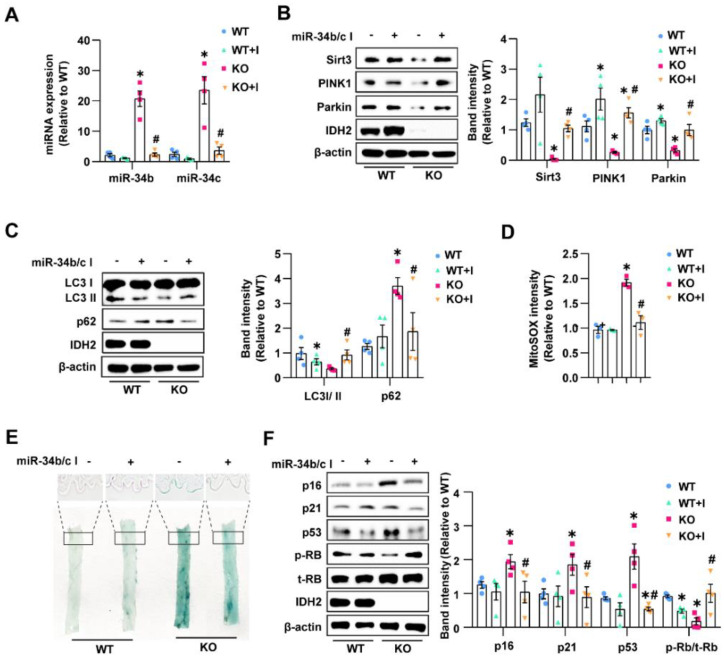
Systemic inhibition of miR-34b/c ameliorated endothelial senescence: (**A**) Abundance of miR-34b or miR-34c in WT or IDH2 KO with or without miR-34b/c inhibitor by qPCR. (**B**) Western blot analysis of Sirt3, PINK1, and Parkin expression. (**C**) Quantification of LC3 II/I ratio and p62 by Western blot. (**D**) mtROS measurement using MitoSOX fluorescence intensity in endothelial cells. (**E**) SA-β-gal staining on endothelium surface from aorta (20×). (**F**) Protein expression analysis of cell cycle regulators using Western blot. *n* ≥ 3 per group, mean ± SEM, * *p* < 0.05 compared with WT without miR-34b/c I, # < 0.05 compared with IDH2 KO without miR-34b/c I.

We investigated whether endothelial miR-34b/c is necessary for repressing Sirt3, PINK1, and Parkin expression in IDH2-knockout mice. Mouse anti-miR-34b/c was administered systemically using osmotic mini-pumps for 2 weeks, resulting in significant suppression of miR-34b/c in mouse endothelial cells (Figure 6A). In vivo, delivery of the miR-34b/c antibody prevented IDH2-knockout-induced downregulation of Sirt3, PINK1, and Parkin and recovered p62, LC3 II/I, and mitochondrial ROS levels (Figure 6B–D). Subsequently, we examined the effect of miR-34b/c inhibition on endothelial senescence. Systemic inhibition of miR-34b/c decreased SA-β-gal positivity on the endothelial surface of IDH2-knockout mice aortas (Figure 6E). It ameliorated IDH2-knockout-induced expression of cell cycle regulators and dephosphorylation of RB in endothelial cells from IDH2-knockout mice (Figure 6F). These results indicated that upregulated miR-34b/c activation accelerated endothelial senescence in IDH2-knockout mice

## 4. Discussion

Vascular cellular senescence is a significant risk factor for cardiovascular diseases in aging adults and causes deterioration of vascular functionality. The underlying mechanisms of endothelial senescence should be examined to develop new therapeutic approaches. In humans, the circulating level of α-KG is reduced during aging, but α-KG administration extends lifespan and reduces morbidity in aged mice and Caenorhabditis elegans [20,21]. α-KG can be produced via two major metabolic pathways. One is generated by the oxidative decarboxylation of isocitrate via IDH2, and another is from the oxidative deamination of glutamate by glutamate dehydrogenase (GDH). In addition, sirt3 has been reported to activate GDH through deacetylation to promote α-KG production. Although glutamine-dependent reductive carboxylation acts as a minor source of α-KG in various normal cells, IDH2 is located in mitochondria and may play the major catalytic role in the isocitrate to α-KG reaction. In addition, a previous study reported that a mutation in the active site of IDH2 induced 2-hydroxyglutarate production instead of α-KG, demonstrating the crucial role of IDH2 in α-KG preservation [22]. However, the expression of α-KG and its modulation by IDH2 have not been investigated in endothelial cells until now. This study showed that α-KG levels were significantly reduced in endothelial cells, correlating with IDH2 inhibition. Therefore, IDH2 deficiency may underlie the decreased α-KG levels and accelerate endothelial senescence. We showed that IDH2 knockout reduced α-KG accumulation, leading to endothelial senescence and SA β-gal activation, cell cycle arrest, and dephosphorylation of RB in vitro and in vivo. In this study, we investigated the underlying mechanism of IDH2 deficiency-induced endothelial senescence.

Mitophagy selectively removes dysfunctional mitochondria and preserves mitochondrial homeostasis. However, impaired mitophagy facilitates damaged mitochondrial accumulation, contributing to cellular senescence and age-related diseases. Since IDH2 downregulation induced mitochondrial dysfunction associated with increased mitochondrial ROS level and mitochondrial fragmentation [9], inhibition of mitophagy may be a possible mechanism of IDH2 deficiency-triggered endothelial senescence. However, mitochondria-localized Sirt3 is also closely associated with cellular senescence and regulation of mitochondrial oxidative stress [23,24]. Sirt3 expression was decreased in senescent bone marrow mesenchymal stem cells and played an essential role in alleviating ROS-induced premature senescence [23]. The present study demonstrated that IDH2 downregulation markedly suppressed mitophagy and reduced Sirt3 expression. Interestingly, PINK1, Parkin, and Sirt3 proteins were reduced in expression, but their mRNA levels were increased, indicating that miRNA regulatory interactions may be associated with inhibited protein synthesis. However, whether the increased mRNA levels of PINK1 and Sirt3 are only carried out by miR-34b/c is ambiguous. The cellular environment is complex, and loss of IDH2 has been reported to impair mitochondrial function and trigger mitoROS, which may also interplay with miRNA-mediated gene expression [10]. Numerous miRNAs act as important regulators of senescence-dependent changes in many cell lines [25]. Lyu et al. found that the age-related generation of miR-29 acts as a key inhibitor during the aging of the H4K20me3, which controls genome stability and promotes cellular senescence [26]. Other studies reported that the miR-34 family suppresses cancer cell growth by inducing cellular senescence and impeding mitophagy flux via site-specific inhibition [17,18,27]. In addition, Aikaterini et al. showed that miR-34b and miR-34c contributed to human arterial atherosclerosis and aging by disrupting Sirt1 [28]. Our results revealed that IDH2 knockout upregulated the miR-34b/c level, and its overexpression led to repression of Sirt3, PINK1, and Parkin, which prevented mitophagy and elevated mitochondrial ROS levels. Systemic inhibition of miR-34b/c using an osmotic pump in IDH2-knockout mice and inhibition of miR-34b/c in HUVECs recovered mitophagy-related protein expression and mitochondrial ROS levels, alleviating endothelial senescence. Therefore, we propose that miR-34b/c is crucial in inhibiting the mitochondrial repair capacity of IDH2-knockout-induced senescent endothelial cells by suppressing mitophagy and elevating mitochondrial ROS levels, thereby promoting endothelial senescence. In addition, transfection of the miR-34b/c mimic in HUVECs also increased the levels of cell cycle regulators (p16, p21, and p53) (Supplementary Figure S1), confirming the role of miR-34b/c in regulating cellular senescence. A previous study reported that IDH2 expression was decreased in senescent mouse embryonic fibroblasts, and IDH2 deletion promoted senescence by inducing ROS generation and cell cycle arrest [29]. Our results suggest that the reduction in IDH2 expression is the leading cause of suppression of the α-KG level in aging endothelial cells. Furthermore, different from the increased level of citrate in siIDH2, the glutamine level in the siIDH2 group is decreased compared to the control group (Supplementary Figure S2). Glutamate generated from glutamine is converted to α-KG by glutamate dehydrogenase. Deletion of IDH2 results in the accumulation of citrate associated with decreased α-KG production. Generally, glutamine can be converted to α-KG to equal the amount of α-KG. However, our results showed that IDH2 deficiency itself caused deprivation glutamine, which failed to supply enough α-KG in the TCA cycle and further contributed to endothelial senescence. Manipulation of the IDH2 and miR-34b/c pathways may provide opportunities for therapeutic modulation of endothelial senescence, thereby improving vascular aging.

There are several limitations to this study. First, endothelial cell-specific knockout of IDH2 may have overestimated the physiologically relevant results compared with IDH2-knockout mice. We are currently generating endothelial-specific IDH2-knockout mice and plan to use them in further research. Second, we did not investigate whether α-KG supplementation rescues mice from IDH2-knockout-induced endothelial cell senescence, which will be investigated in further studies.

## 5. Conclusions

**Figure 7:**
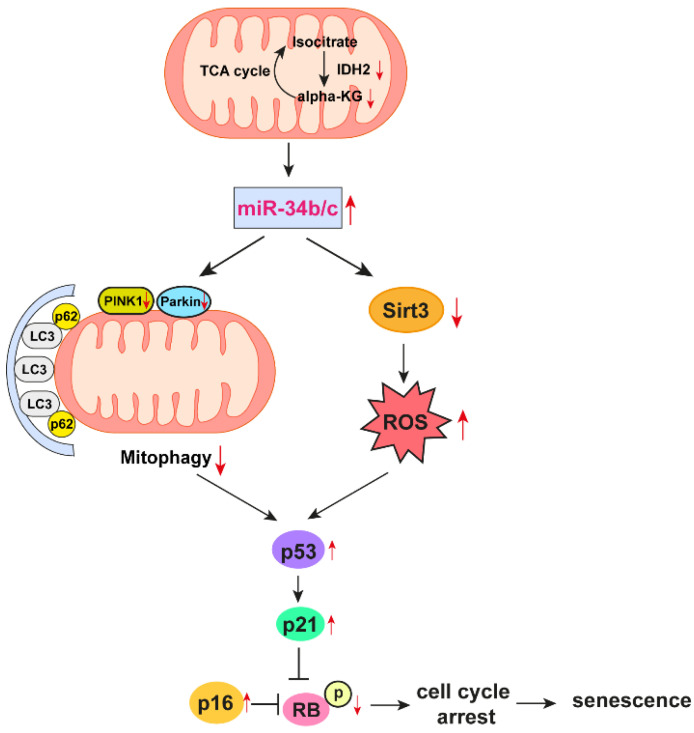
Schematic representation of the proposed pathway of endothelial senescence induction in IDH2 deficiency condition.

In summary, this study demonstrates that IDH2 deficiency is critical for the suppression of α-KG levels, which induced endothelial senescence via miR-34b/c inhibition of mitophagy and mitochondrial ROS elevation in endothelial cells (Figure 7). Therefore, the IDH2/α-KG/miR-34b/c axis may provide a potential therapeutic target for age-related vascular diseases.

## Data Availability

The data are available within the article and Supplementary Materials.

## Supplementary Materials

The following supporting information can be downloaded at: https://www.mdpi.com/article/10.3390/antiox12030585/s1, Table S1: Primer sequence for RT-PCR analysis; Figure S1: Induction of miR-34b/c increased p16, p21, and p53 quantified by Western blot; Figure S2: Relative intracellular levels of citrate and glutamine; Figure S3: Representative micrographs of MitoSox Red-labeled HUVECs; Figure S4: Sequence matching of miRNA-target mRNA interaction.
