# Supplementary material for: IDH2 Deficiency Promotes Endothelial Senescence by Eliciting miR-34b/c-Mediated Suppression of Mitophagy and Increased ROS Production
Source: Antioxidants (Basel). 2023 Feb 27;12(3):585. doi: 10.3390/antiox12030585 (PMC10045915; doi:10.3390/antiox12030585)
Supplement: Supplementary file 1 [file antioxidants-12-00585-s001.zip › antioxidants-2224401-supplementary.pdf]

Supplementary Table. 1

Gene	Forward Sequence (5'-3')	Reverse Sequence (5'-3')	Species
Sirt3	GCAGCTCCCAGTTTCTTCTTT	CACCATGACCACCACCCTCT	Human
PINK1	TCTAAGCCTCTGGGGTGAACA	AGCGTTTCACACTCCAGGTT	Human
Parkin	GTGTTTGTCAGGTTCAACTCCA	GAAAATCACACGCAACTGGTC	Human
Sirt3	TGCTTCTGCGGCTCTATACAC	GGTTTCACAACGCCAGTACAG	Mouse
PINK1	GCAATGCCGCTGTGTATGAAG	TGGAGGAACCTGCCGAGATAT	Mouse
Parkin	GGCTGCGGGTTTGTTTTCTG	GGCTTGGTGGTCTTCTTGATG	Mouse
miR-34b-3p	CAATCACTAACTCCACTGCCATAA	-	Human
miR-34b-5p	CGCTAGGCAGTGTCATTAGCTG	-	Human
miR-34c-3p	CTAACCACACGGCCAGGA	-	Human
miR-34c-5p	CAGGCAGTGTAGTTAGCTGATTG	-	Human
miR-34b-3p	CACTAACTCCACTGCCATCAAA	-	Mouse
miR-34b-5p	GGCAGTGTAATTAGCTGATTGTAAA	-	Mouse
miR-34c-3p	CTAACCACACAGCCAGGAAAA	-	Mouse
miR-34c-5p	CAGGCAGTGTAGTTAGCTGATTG	-	Mouse

Supplementary Figure S1

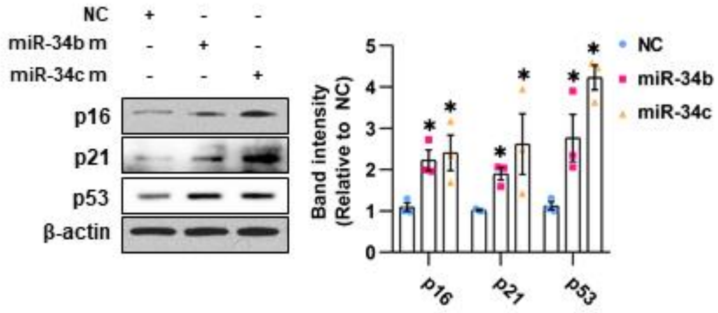

Figure S1. Western blot analysis of p16, p21, and p53 in HUVECs with NC or miR-34b m or miR-34c m. n≥3 per group, mean ± SEM, *P < 0.05 compared with NC group.

Supplementary Figure S2

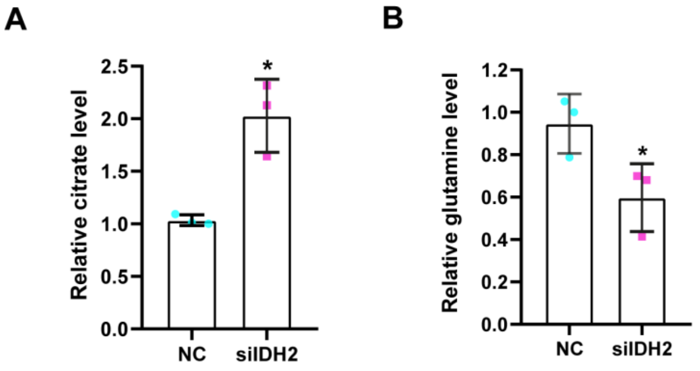

Figure S2. Relative intracellular levels of citrate (A), and glutamine (B) in HUVECs cells following treatment with NC or siIDH2. n≥3 per group, mean ± SEM, *P < 0.05 compared with NC group.

Supplementary Figure S3

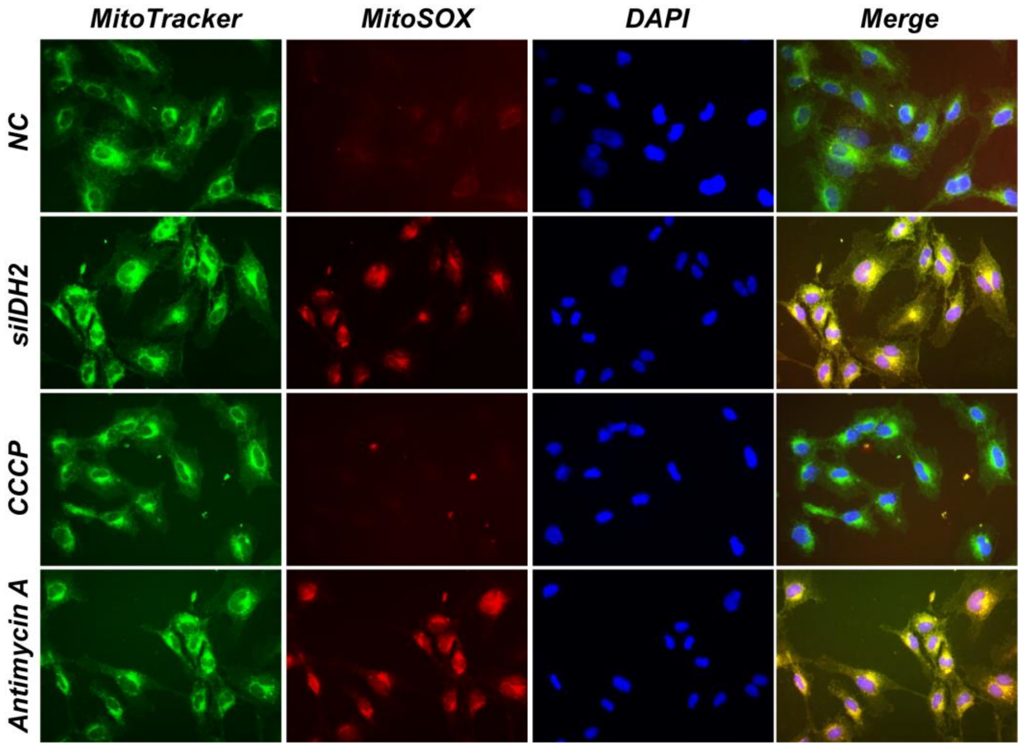

Figure S3. Representative micrographs of MitoTracker Green- and MitoSox Red-labeled HUVECs in the NC (Control) or siIDH2 (100 pmol) or CCCP (20 μM, negative control) or Antimycin A (5 μM, positive control) ; blue shows nuclei staining with DAPI.

Supplementary Figure S4

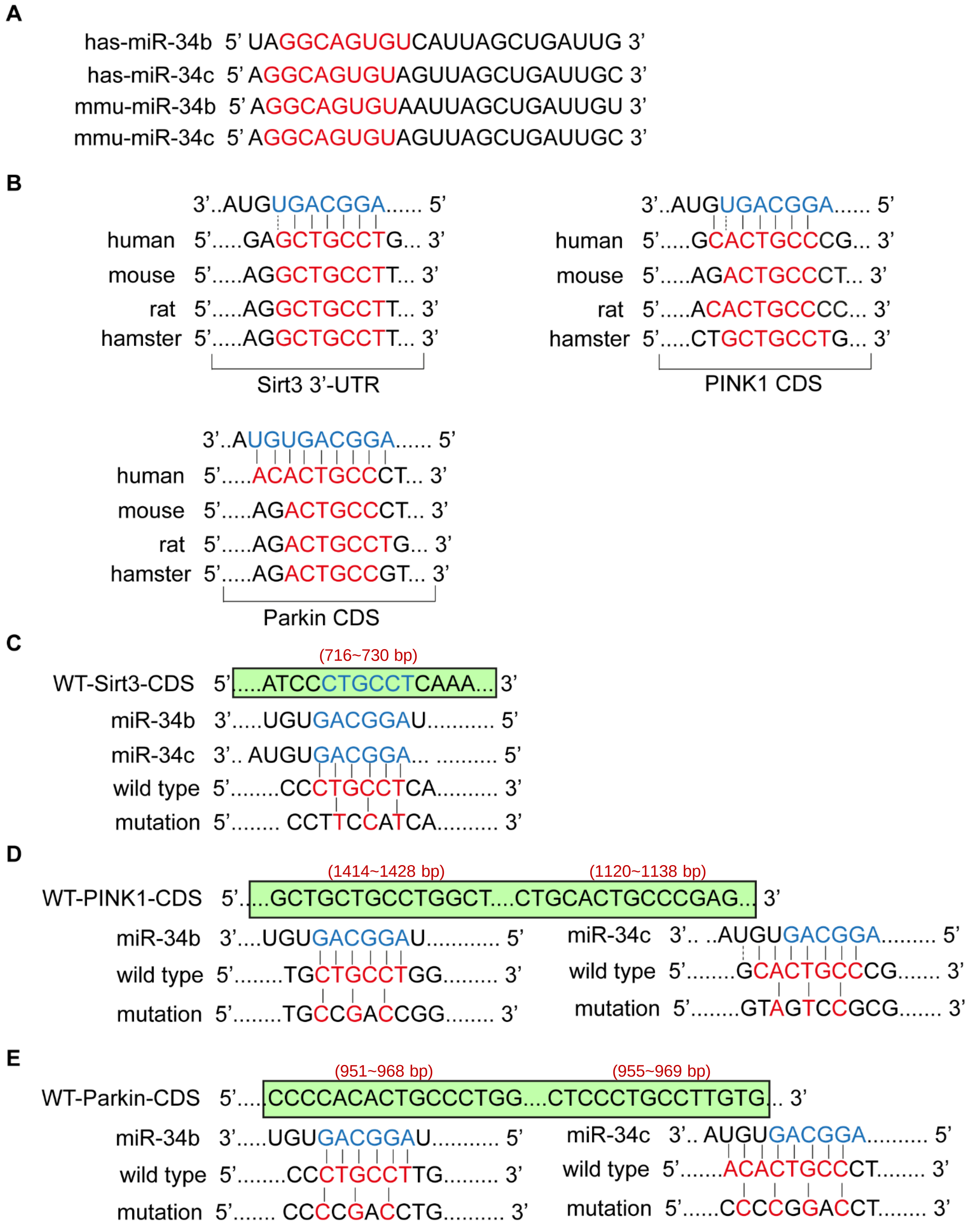

Figure S4. (A) Sequence and seed site of human and mouse miR-34b/c. (B) Representative mode of miR-34b/c targeting the 3’-UTR or CDS region of Sirt3, PINK1, and Parkin. (C, D, E) Sequence mutation sites of Sirt3, PINK1, and Parkin CDS region.
